# Concordance and determinants of mothers’ and children’s diets in Nigeria: an in-depth study of the 2018 Demographic and Health Survey

**DOI:** 10.1136/bmjopen-2022-070876

**Published:** 2023-07-11

**Authors:** Nadia Akseer, Hana Tasic, Olutayo Adeyemi, Rebecca Heidkamp

**Affiliations:** 1 International Health, Johns Hopkins University Bloomberg School of Public Health, Baltimore, Maryland, USA; 2 Modern Scientist Global, St Catharines, Ontario, Canada; 3 Department of Human Nutrition and Dietetics, University of Ibadan, Ibadan, Nigeria

**Keywords:** nutrition, nutrition & dietetics, public health, community child health

## Abstract

**Abstract:**

**Objectives:**

Improving the diversity of the diets in young children 6–23 months is a policy priority in Nigeria and globally. Studying the relationship between maternal and child food group intake can provide valuable insights for stakeholders designing nutrition programmes in low-income and middle-income countries.

**Design:**

We examined the relationship between maternal and child dietary diversity among 8975 mother–child pairs using the Nigeria 2018 Demographic and Health Survey (DHS). We assessed concordance and discordance between maternal and child food group intake using the McNemar’s χ^2^ test, and the determinants of child minimum dietary diversity (MDD-C) including women MDD (MDD-W) using hierarchical multivariable probit regression modelling.

**Setting:**

Nigeria.

**Participants:**

8975 mother–child pairs from the Nigeria DHS.

**Primary and secondary outcome measures:**

MDD-C, MDD-W, concordance and discordance in the food groups consumed by mothers and their children.

**Results:**

MDD increased with age for both children and mothers. Grains, roots and tubers had high concordance in mother–child dyads (90%); discordance was highest for legumes and nuts (36%), flesh foods (26%), and fruits and vegetables (39% for vitamin-A rich and 57% for other). Consumption of animal source food (dairy, flesh foods, eggs) was higher for dyads with older mothers, educated mothers and more wealthy mothers. Maternal MDD-W was the strongest predictor of MDD-C in multivariable analyses (coef 0.27; 95% CI 0.25 to 0.29, p<0.000); socioeconomic indicators including wealth (p<0.000), mother’s education (p<0.000) were also statistically significant in multivariable analyses and rural residence (p<0.000) was statistically significant in bivariate analysis.

**Conclusion:**

Programming to address child nutrition should be aimed at the mother–child dyad as their food consumption patterns are related and some food groups appear to be withheld from children. Stakeholders including governments, development partners, non-governmental organizations, donors and civil society can act on these findings in their efforts to address undernutrition in the global child population.

**STRENGTHS AND LIMITATIONS OF THIS STUDY**

- Analysis is done using a dataset of large sample size.
- This is one of the first assessments of the relationship between maternal and child diet diversity scores using newly available minimum dietary diversity in women.
- The data used are from a cross-sectional survey, which can produce inferences about correlation but not causation.
- Comparable food groups for mothers and their children were generated, though categories were slightly different in the original dataset.
- The data used are collected through 24-hour recalls, which may be susceptible to recall bias, and do not consider seasonality.

## Introduction

 The period between 6 and 23 months of age is characterised by rapid growth and, particularly for children in low-resources contexts, increased exposure to infectious diseases. Continued breast feeding with gradual introduction of diverse, nutrient dense complementary foods is recommended to meet elevated nutritional needs in this age group.^1^,^3^

Compared with other household members, infants in low-income and middle-income countries (LMICs) are more likely to consume monotonous cereal-based diets that are of lower nutrient density.^3 4^ Studies in LMICs have demonstrated a strong positive relationship between maternal and child dietary diversity.^5^,^7^ Some studies show withholding of specific foods from children such as fish, meat and leafy green vegetables.^58^,^10^ Other studies have found preferential allocation of animal source foods to children compared with mothers, particularly in the case of milk and milk products.^11 12^ However, few studies examining the relationship between maternal child diet quality use nationally representative data.

Minimum dietary diversity (MDD) is a simple dichotomous indicator developed for inclusion in large-scale household surveys. MDD in children 6–23 months (MDD-C) is defined as a minimum of five of eight food groups consumed in the previous 24 hours.^13^ MDD in women of reproductive age (MDD-W) is defined as a minimum of 5 of 10 total food groups consumed in the previous 24 hours.^14^ MDD-C includes breastmilk as a food group, while the MDD-W classifies dark leafy green vegetables and nuts and seeds as separate food groups. The two indicators have been validated against population-appropriate measures of nutrient density.^15^

MDD-C^16^ has been collected as part of the core questionnaires for the Demographic and Health Surveys (DHS) and the Multiple Indicator Cluster Surveys since 2008. MDD-W was only added to the DHS core questionnaire in round 8 which started field-based data collection in late 2019.^17^ At the time of our analysis, DHS-8 recoded datasets were not yet publicly available.^18^ However, Nigeria included MDD-W in the country’s 2018 DHS,^19^ a round 7 survey, which allows for examination of the relationship between maternal and child diet using nationally representative data. This paper intends to explore the relationship between maternal and child diet diversity to inform policies that can improve diet diversity of children in the critical period of 6–23 months of age.

## Methods

In our analysis, first, we estimate individual food group intake, MDD-W and MDD-C for Nigerian mothers and their children 6–23 months; second, we estimate concordance and discordance in the food groups consumed by mothers and their children, and present analyses by child age, maternal age, education and wealth. Third, to identify factors associated with MDD-C, we estimate the crude and covariate adjusted effect of MDD-W on MMD-C and used an evidence-based conceptual framework to examine MDD-W relative to other known determinants of MDD-C.

### Data source and design

The 2018 Nigeria DHS (NDHS) used a stratified, two-stage cluster design. Enumeration areas (EAs) were the sampling units for the first stage, and the second stage consisted of a complete listing of households in each of the 1400 EAs that were selected in stage 1. The final national sample included around 42 000 households. With a response rate of 99%, 42 121 women aged 15–49 were identified for individual interviews.^20^ The 2018 NDHS questionnaire included, among other questions, women’s and children’s feeding practices, nutritional status of women and children, adult and childhood mortality, and a variety of household characteristics including wealth index, and head of household characteristics.

### Outcomes

Our analyses were restricted to pairs of mothers aged 15–49 years and their children aged 6–23 months, for a total of 8975 dyads. MDD-C is the per cent of children 6–23 months old who ate five or more food groups out of a possible eight in the previous 24 hours.^21 22^ The eight food groups are: (1) breastmilk, (2) grains, roots and tubers, (3) legumes and nuts, (4) dairy products (infant formula, milk, yoghurt, cheese), (5) flesh foods (meat, fish, poultry and liver/organ meats), (6) eggs, (7) vitamin A rich fruits and vegetables and (8) other fruits and vegetables. MDD-W is the per cent of women 15–49 years old who have consumed five or more of the following food groups in the previous 24 hours: (1) grains, white roots and tubers, and plantains, (2) pulses (beans, peas and lentils), (3) nuts and seeds, (4) dairy, (5) meat, poultry and fish, (6) eggs, (7) dark green leafy vegetables, (8) other vitamin A-rich fruits and vegetables, (9) other vegetables and (10) other fruits.^14^

### Covariates

Using a modified version of the UNICEF framework,^23^ we categorised determinants of dietary intake available in the NDHS 2018 dataset into proximal (immediate), intermediate (underlying) and distal (basic) levels (see online supplemental figure 1). The distal determinants include wealth index and maternal education reflecting inadequate financial capital and household access to education. Intermediate causes include urban or rural residence reflecting sociocultural context and household-related factors including sex of household head, women’s decision-making and the total number of people living in the household. Proximal-level determinants of dietary intake include individual-level factors such as child age, child sex, maternal age and parity.

### Statistical analysis

We calculated the proportion of children and women consuming each food group and reaching MDD at the national level and by age group (15–19, 20–29, 30–39, 40–49 years of age for women, and 6–11, 12–23 months of age for children) with 95% confidence intervals (CIs).

We analysed concordance and discordance between a mother–child dyad’s consumption of food groups using the following seven common food groups: (1) grains, roots, tubers, (2) legumes and nuts, (3) dairy, (4) flesh foods, (5) eggs, (6) vitamin A rich fruits and vegetables and (7) other fruits and vegetables. To create common categories using maternal data we combined pulses and nuts and seeds with the legumes and nuts category; dark green leafy vegetables with other vitamin A rich fruits and vegetables; and other fruits with other vegetables. We produced estimates for four subcategories: concordance (A) both mother and child consumed or (B) both did not consume; and discordance or (C) mother consumed and child did not or (D) child consumed and mother did not. The concordance and discordance analyses were stratified to explore effect modification by child age (6–11, 12–23), maternal age (15–19 and 35+), urban/rural residence, maternal education (none, any) and wealth index (bottom two wealth quintiles, top three wealth quintiles). We tested statistical differences using McNemar’s χ^2^ test and displayed significance at three levels: p<0.05, p<0.01, p<0.001.

We used hierarchical multivariable probit regressions to estimate the crude and adjusted effect of MDD-W on her child’s individual food group intake and MDD-C. We adjusted models hierarchically for distal, intermediate and proximal confounders of the relationship between maternal and child diet.^24^ A two-level hierarchical modelling was conducted first adjusting for distal and intermediate factors (wealth index, maternal education and urban or rural residence, ie, adjusted level 1 estimates), followed by proximal factors (age, child sex, maternal age, and parity, ie, adjusted level 2 estimates).

To understand the relative contribution of various risk factors and determinants to MDD-C, we fit additional hierarchical probit regressions. Using a stepwise approach, we first modelled distal-level factors (wealth index and maternal education) and used a threshold of p<0.15 in bivariate analyses for selecting variables for the multivariable model. Next, we did the same for intermediate-level variables (rural residence, number of household members, sex of household head, and women’s decision-making (variable from 0 to 3 based on whether the respondent participates in decisions on own healthcare, large household purchases, and visits to family and relatives), and then for proximate level variables (MDD-W, child age, child sex, maternal age, parity). Finally, we applied backward elimination for each of the distal, intermediate and proximal levels to select variables that were significant at p<0.05. We produced predicted probabilities of each factor on MDD-C with 95% CIs.

We accounted for the complex survey design and sampling weights and used Stata SE V.15.1 for all analyses. We considered a type 1 error rate of alpha=0.05.

### Patient and public involvement

Patients and the public were not involved in the design, conduct, reporting, or dissemination plans of our research as we conducted a secondary analysis of data that were already collected.

## Results

We analysed data from 8975 mother–child pairs (table 1). The mean age of children was 14 months old, and 28 years of age for mothers. Over 44% of mothers had no education, while 32% had a secondary level of education. Over 60% lived in rural areas and the mean household size was 6.7 people.

**Table 1:** Characteristics of study sample

	Mean/SD or freq/%
Child characteristics, n=8975	
Age, months	13.9 months (SD=5.0)
Age group	
6–11 months	3109, 34.6%
12–23 months	5886, 65.4%
Female	4364, 48.6%
Maternal characteristics, n=8975	
Age, years	28.4 (SD=6.7)
Age group	
15–19 years	Freq=603, 6.7%
20–29 years	Freq=4542, 50.6%
30–39 years	Freq=3218, 35.9%
40–49 years	Freq=612, 6.8%
Education	
None	Freq=3977, 44.3%
Primary	Freq=1274, 14.2%
Secondary	Freq=2970, 33.1%
Higher	Freq=754, 8.4%
Parity	3.88 (SD=2.56)
Household characteristics, n=8975	
Rural residence	Freq=5499, 61.3%
Wealth quintile	Poorest=21.9% (1965)
	Poorer=22.0% (1975)
	Middle=20.6% (1849)
	Richer=18.7% (1674)
	Richest=16.8% (1512)
Women head of household	Freq=769, 8.6%
Household size	6.8 (SD=3.7)
No of children	2.1 (SD=1.2)
Child diet diversity n=8975	
# food groups consumed (max: 8)	3.30 (SD=1.62)
MDD-C (≥5 groups)	1990, 22.2%
Maternal diet diversity n=8975	
# food groups consumed (max: 10)	4.69 (SD=1.81)
MDD-W (≥5 groups)	4538, 50.6%

MDD-C, minimum dietary diversity for children; MDD-W, MDD for women.

**Figure 1:**
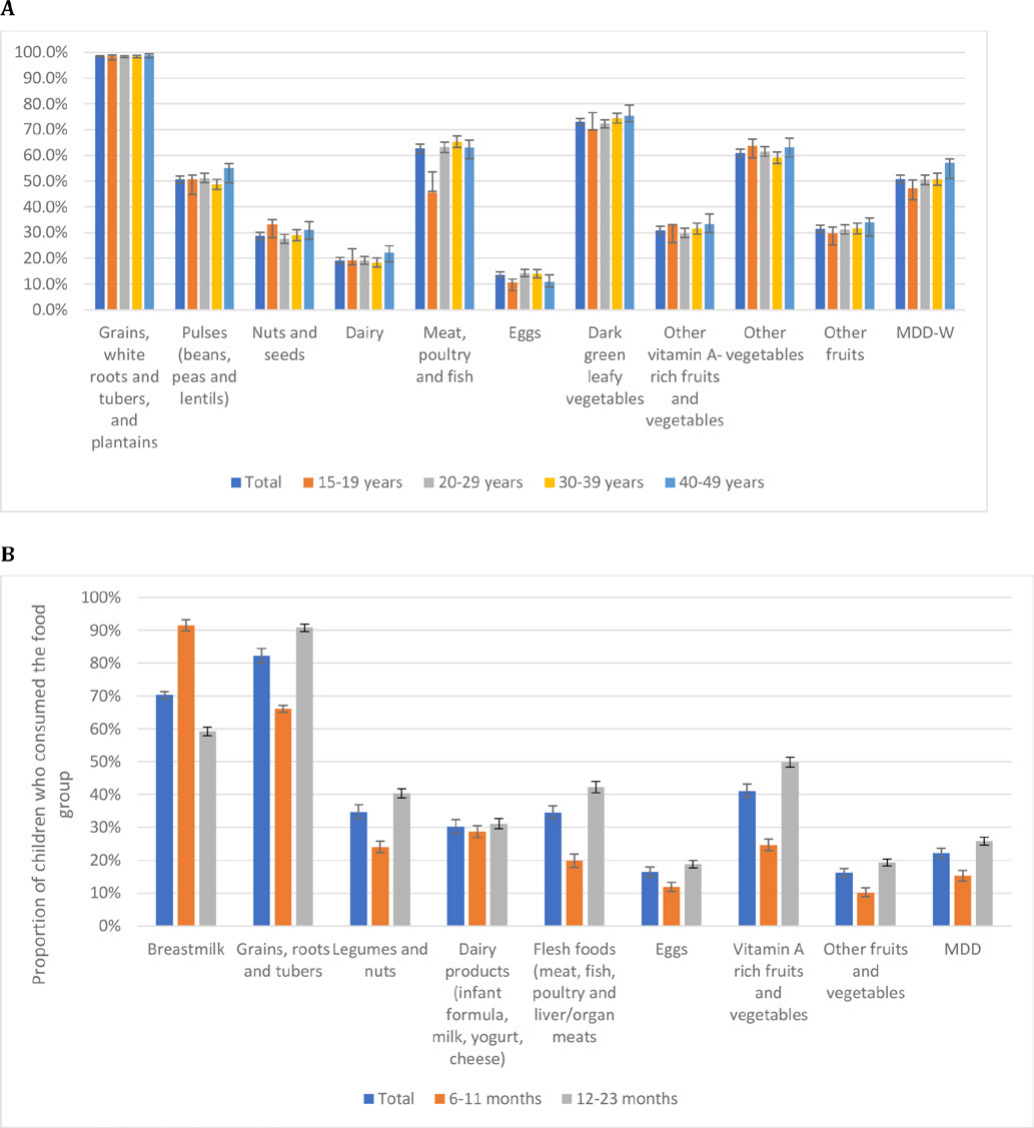
(**A**) Percentage of mothers consuming different food groups in past 24 hours and achieving MDD-W by age group. (**B**) Percentage of children consuming different food groups in past 24 hours and achieving MDD-C by age group. MDD-C, child minimum dietary diversity; MDD-W, women MDD.

Approximately 22% of children achieved MDD-C and over 50% of mothers achieved MDD-W. Figure 1A,B shows the proportions of mothers and children achieving their respective MDD and consuming different food groups disaggregated by age. Youngest mothers (15–19 years) had the lowest MDD-W (47%) and the oldest mothers (40–49 years) had the highest (57%). This trend was significant for two food groups: dark green leafy vegetables and other fruits. The consumption of pulses and meat, poultry, and fish was generally higher for older mothers. Notably, a smaller proportion of mothers aged 15–19 consumed flesh foods (46%) compared with all other age groups, with a difference of over 17 percentage points compared with oldest mothers (figure 1A).

A higher proportion of children aged 12–23 months achieved MDD-C (26%) compared with those aged 6–11 months (15%) (figure 1B). For all food groups apart from breastmilk, consumption was higher among children in the older age group than those in the younger age group. Notable differences by age are observed for consumption of legumes and nuts, flesh foods, and vitamin A rich fruits and vegetables.

**Figure 2:**
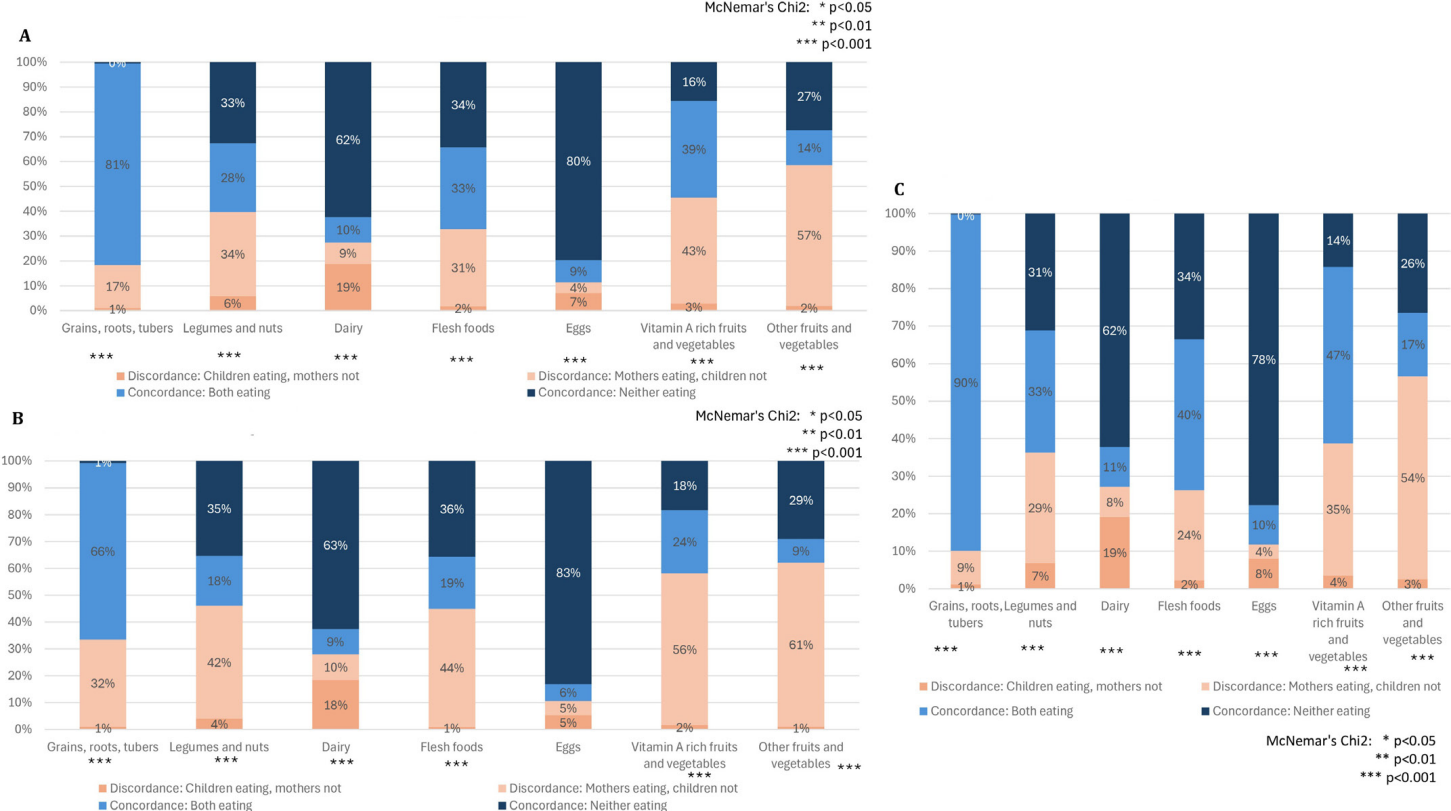
(**A**) Percentage concordance and discordance between maternal and child consumption of specific food groups in past 24 hours. (**B**) Percentage concordance between maternal and child consumption of specific food groups in past 24 hours (child age 6–11 months). (**C**) Percentage concordance between maternal and child consumption of food groups (child age 12–23 months).

Maternal–child dietary concordance was highest for grains, roots, and tubers (90%), and eggs (88%) (figure 2A). Discordance was highest for other fruits and vegetables; among 54% of pairs, only mother consumed the food group compared with 3% with only child. Aside from dairy and eggs, discordance favoured the mother (ie, mothers consumed more) for all other food groups (figure 2A). Among the younger child age (6–11 months), high discordance was evident for all food groups except dairy (range 10%–82%) (figure 2B). Discordance was generally lower for children 12–23 months old (figure 2C), but mothers were more likely than children to consume grains, roots and tubers (9%), flesh foods (24%), legumes and nuts (29%), vitamin A rich fruits and vegetables (35%) and other fruits and vegetables (54%).

**Figure 3:**
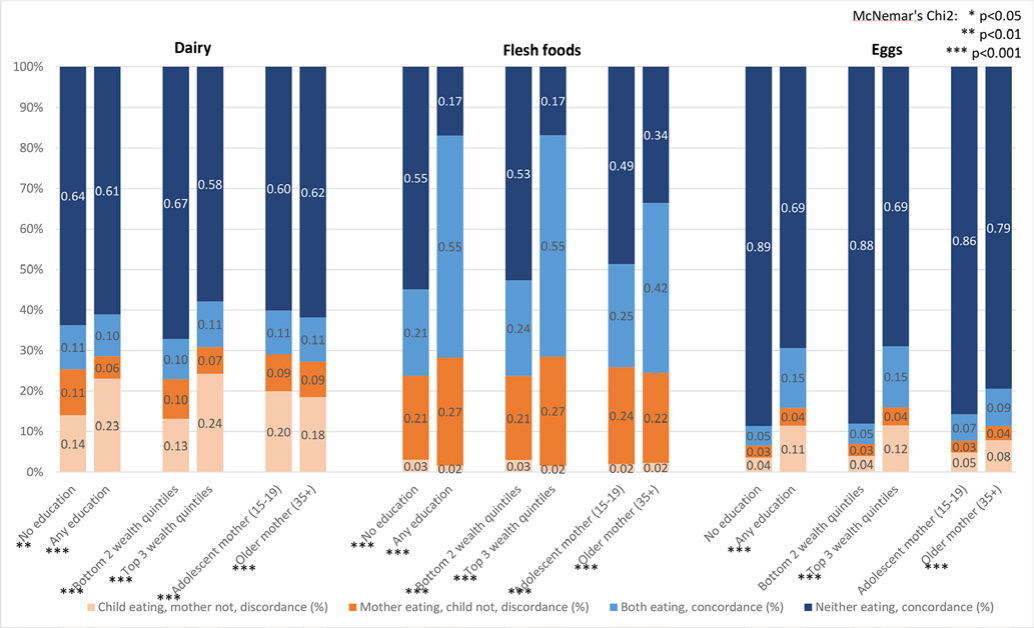
Percentage concordance and discordance between maternal and child consumption of animal source foods in past 24 hours by education, wealth and mother’s age.

Online supplemental figures show concordance and discordance for all groups disaggregated by subgroups including by maternal age (online supplemental figure 2A,B), urban/rural residence (online supplemental figure 3A,B), maternal education (online supplemental figure 4A,B) and wealth quintiles (online supplemental figure 5A,B). Online supplemental figure 6 shows the concordance and discordance between mothers and children with regard to MDD. Generally, discordance patterns did not vary by subgroups, but concordance did. Younger mothers (15–19 years) and their children had a tendency to eat less flesh foods (49% concordance for neither consuming) compared with other mothers (35+ years) (34% concordance). Similarly, among mothers in rural areas, the uneducated and the poorest, neither mother nor child were as likely to consume animal source proteins (ie, flesh food and eggs). Rates of concordance and discordance for consumption of animal source food categories by select subgroups are presented in figure 3.

There was a minimal or small effect of MDD-W on child consumption of two groups: breastmilk and grains, roots, and tubers (online supplemental figure 7A,B). In both crude and confounder-adjusted analyses, maternal MDD-W had significant positive association with child consuming legumes and nuts (23% increased probability of child consuming if the mother achieved MDD-W, dairy (17% increase), flesh foods (17% increase), eggs (15% increase), vitamin A rich fruits and vegetables (22% increase), other fruits and vegetables (17% increase) and overall MDD-C (27% increase) (online supplemental figure 7C–I).

In both crude (bivariate) and adjusted (multivariable) analyses, wealth and maternal education are significant determinants of MDD-C, with those in richer and richest wealth quintiles having a greater predicted probability of achieving MDD-C, and higher maternal education associated with greater predicted probability of achieving MDD-C compared with no maternal education (table 2). Rural residence and larger household size were both negatively associated with MDD-C in bivariate analyses. However, none of the intermediate-level variables analysed were significantly associated with MDD-C in the multivariable analysis. Women’s decision-making was positively associated with MDD-C in bivariate analysis, but sex of household head was not. Of the proximal-level variables, after adjusting for confounders in multivariable analyses, having a mother achieve MDD-W conferred a greater predicted probability of a child achieving MDD-C, as did older child age.

**Table 2:** Multivariable adjusted drivers of MDD-C (n=8975)

	Categories	Bivariate			Multivariable		
		Predicted probability	95% CI	P value	Predicted probability	95% CI	P value
Distal variables							
Wealth (n=8975)	Poorest	REF			REF		
	Poorer	0.00	−0.03 to 0.02	0.837	−0.01	−0.04 to 0.02	0.390
	Middle	0.03	0.00 to 0.06	0.048	0.00	−0.03 to 0.03	0.993
	Richer	0.11	0.07 to 0.15	0.000	0.05	0.01 to 0.09	0.011
	Richest	0.20	0.16 to 0.24	0.000	0.10	0.06 to 0.15	0.000
Maternal education (n=8975)	None	REF			REF		
	Primary	0.04	0.01 to 0.07	0.007	0.03	−0.01 to 0.06	0.107
	Secondary	0.12	0.09 to 0.14	0.000	0.08	0.05 to 0.10	0.000
	Higher	0.25	0.20 to 0.30	0.000	0.16	0.10 to 0.21	0.000
Intermediate variables							
Rural residence (n=8975)	Urban	REF					
	Rural	−0.10	−0.13 to −0.08	0.000	NS		0.162
No of household members (n=8975)	Continuous	−0.01	−0.01 to −0.00	0.000	NS		0.560
Sex of household head (n=8975)	Male	REF					
	Female	0.02	−0.01 to 0.06	0.245	NS		
Women’s decision-making (n=8519)	Continuous	0.02	0.02 to 0.03	0.000	NS		0.769
Proximal variables							
MDD-W (n=8975)	No	REF					
	Yes	0.29	0.27 to 0.32	0.000	0.27	0.25 to 0.29	0.000
Child age (n=8975)	Continuous	0.01	0.01 to 0.01	0.000	0.01	0.01 to 0.01	0.000
Child sex (n=8975)	Male	REF					
	Female	0.00	−0.02 to 0.02	0.963	NS		
Maternal age (n=8975)	Continuous	0.00	0.00 to 0.00	0.002	NS		0.334
Parity (n=8975)	Continuous	−0.01	−0.01 to−0.00	0.002	NS		0.699

MDD-C, minimum dietary diversity for children; MDD-W, MDD for women; NS, not significant.

## Discussion

Our analysis of diet diversity among mother–child dyads from a large nationally representative sample in Nigeria reveals several important insights. Similar to findings from other LMIC contexts,^5^,^7^ we found strong relationships between the mother and child’s dietary diversity, particularly among older children 12–23 months as well as a relationship between MDD-W and child consumption of most individual food groups. Exceptions include breastmilk and grains, roots and tubers, which children consume irrespective of maternal diet. Socioeconomic indicators such as wealth, mother’s education, and rural residence are among the top drivers of child diet diversity, but the mother’s own diet diversity is the strongest determinant of MDD-C.^5^,^7^

We found higher overall dietary diversity among mothers relative to children and evidence of specific food groups that appear be available to the mother but not fed to children including animal source foods and fruits and vegetables. These findings may prove useful to programme planners who are developing infant and young child feeding (IYCF) interventions.

Amugsi *et al* also found higher dietary diversity for mothers compared with children in Ghana suggesting mothers feed children a subset of what is available in the household.^7^ Reasons for discrepancies in maternal and child consumption of particular foods may include beliefs around appropriateness of certain foods for children, (eg, belief that vegetables are hard to digest or belief that meat and eggs are eaten in such small quantities that they are not filling and so dispensable),^7 11 25 26^ that food restriction for children can build a child’s moral character, and other food beliefs and taboos.^725 27^,^29^ There may very well be other determinants which require further study such that the mother is exposed to a different food environment than the child, (eg, by working outside of the home).

Many of our other findings are also corroborated by other studies in and beyond Nigeria. We found that achieving MDD was associated with age for children which is consistent with the global practice of gradually introducing complementary foods as the child ages. Jones *et al* ^30^ found similar findings in Cambodia, Haiti, Kenya, Uganda and Zambia. We also found that dietary diversity of mothers was lowest for adolescent mothers (age 15–19 years) and improved with age—a novel finding for the African context that has important policy implications for adolescent health and nutrition. A study in rural South-West Nigeria found that child age and maternal age were significant determinants of children’s dietary diversity.^31^

This is the first study looking at patterns of food group concordance and discordance between mothers and their children in Nigeria. We found that concordance was high for grains, roots and tubers in Nigeria. Nguyen *et al* also found agreement for consumption of such staple foods in Bangladesh, Ethiopia and Vietnam.^5^ In line with our findings, they also found high discordance for flesh foods, dairy and fruits and vegetables.

For Nigeria, these findings have critical policy implications. First, it appears that some nutrient-rich foods are available to members of the household and yet not fed to the children. This suggests that food access is not always the limiting issue for child dietary diversity, and that the factors related to maternal nutrition knowledge and cultural practices should be further studied.

Moreover, mothers are often not the only caregivers for young children. A considerable per cent of mothers in the 2018 NDHS (70%) were employed at the time of the survey. Mothers eating one or more meals outside the home when they are not with their children could contribute to discordance in maternal and child dietary diversity. The 2018 NDHS did not report the per cent of women who worked outside the home nor who the primary caregiver of children was, but other authors have emphasised the role that other household members play in caring for and feeding young children.^32^,^35^

Thus, policy-makers and donors should consider mass education and social and behaviour change communication (SBCC) approaches that target appropriate IYCF practices so that nutrition knowledge is improved among the population generally, and not just among mothers. Interventions that have targeted other family members in behaviour change communication have reported larger effects than interventions that target mothers only.^36 37^ The WHO also recommends involving other family members in maternal and child nutrition interventions.^38^

For consumption of animal source food (dairy, flesh foods, eggs), we found to have slightly lower overall concordance between mother–child dyads with older mothers, educated mothers and those living in wealthier households. While it was generally more likely in these subgroups that both mother and child consumed the food group (ie, concordance), there were food groups for which discordance was more likely than in reference groups. This pattern is most pronounced for dairy and eggs where wealthier households appear to preferentially give the foods to children over mother; the opposite pattern holds for flesh foods which have higher consumption by mothers.

In a study in Imo State, Nigeria, meat consumption was associated with income, age and education.^39^ These relationships were also evident in our regression analyses as well as an association between maternal education and both maternal and child dietary diversity. Nguyen *et al* found that socioeconomic status was only associated with maternal dietary diversity across Bangladesh, Ethiopia and Vietnam, however, our study found that wealth index was associated with MDD-C.^5^

Numerous studies have explored dietary diversity in Nigeria and have found ethnic and zonal variation as well as a positive relationship with total family income.^40^,^43^ Higher income, education and non-farm enterprise engagement were associated with adequate nutrient intake, and dietary diversity was positively associated with the likelihood of adequate intake.^44^ One study conducted in Lagos, Nigeria found that marital status, maternal level of education, level of complementary feeding knowledge and monthly income were not associated with child dietary diversity, however, that appropriate complementary feeding practices were associated with higher levels of mothers’ education and occupation.^45^

Overall dietary diversity is low in Nigeria, and a substantial proportion of households suffer deficiency of calories, protein and certain micronutrients, particularly in the postplanting season.^41^,^4346^ Complementary feeding knowledge and practices have also been found to be low.^45^ Nigerian children with low dietary diversity score were more likely to be stunted.^40^ This suggests that further exploration of the relationship between maternal and child dietary diversity is necessary using subnational data collected across seasons.

Considering the strong link between maternal and child dietary diversity, programmes aiming to improve child nutrition in Nigeria can include social and behaviour change interventions that target both maternal and child dietary diversity as a dyad.^5 6^ In addition, since discrepancies between consumption of foods exists in mother–child dyads, interventions can include strategies that focus on feeding children as diverse a diet as the mother eats and emphasise the significance of feeding a child all family foods.^5 7^

Specific food taboos need to be addressed. A study in Kaduna state found that although eggs are widely available and affordable, they were not frequently given to children. A demand creation campaign aimed at increasing egg consumption was undertaken.^47^ There is also a need to improve overall MDD in both women and children in Nigeria. Production diversity, enhanced homestead food production activities, nutrition education, production of vegetables and livestock ownership have all been linked to higher child and maternal dietary diversity.^48^,^52^ Our findings also support previous literature on the importance of socioeconomic status, including wealth and education level as important determinants of MDD-C.^5 7^ Programming that addresses these more distal determinants may also contribute to the improved diet diversity across populations.

This study has several notable limitations. While the sample size was large and powered for all analyses, the data is from a cross-sectional survey and at best produces inferences about correlation and not causation. While we were able to combine and produce comparable food groups for mothers and their children, the underlying data were in fact collected with slightly different measures for select food groups, and thus our collapsed food groups may have misclassification. Given the use of 24-hour recalls, the food group markers may be susceptible to recall bias. Our group’s previous studies show that MDD-C in DHS surveys is subject to seasonality,^53^ therefore, its worthwhile conducting an assessment to understand these relations across seasons in Nigeria.

## Conclusion

Findings from this study can guide policy and programming efforts to improve child and maternal diets among vulnerable populations in Nigeria and other similar contexts. Analyses can be replicated in Nigeria over time to assess impacts of policy and contextual changes as well as in other LMICs as national surveys with MDD-W increasingly become available.

Maternal and child dietary diversity are related in Nigeria and programmes aimed at improving IYCF should take this relationship into account. Discrepancies between maternal and child consumption patterns of highly nutritious food groups including legumes and nuts, flesh foods, fruits and vegetables need to be addressed. Programming should encourage children to be fed all the household foods to ensure dietary diversity. Specific food taboos need to be targeted as should poverty and other underlying causes of poor diet in children and women.

## Supplementary material

10.1136/bmjopen-2022-070876 online supplemental file 1

## Data Availability

Data are available in a public, open access repository.
