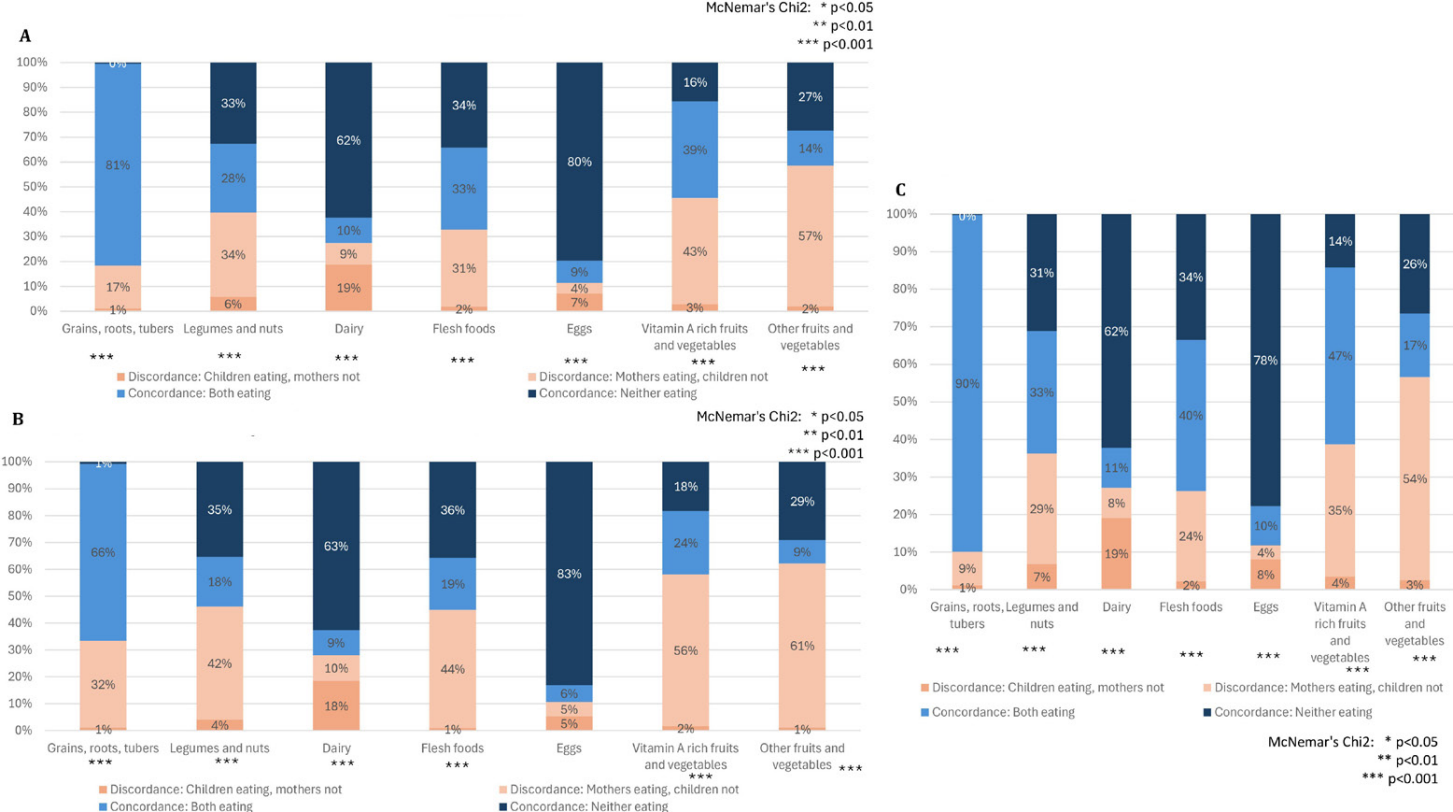# Correction: Concordance and determinants of mothers’ and children’s diets in Nigeria: an in-depth study of the 2018 Demographic and Health Survey

**DOI:** 10.1136/bmjopen-2022-070876corr1

**Published:** 2025-06-09

 Akseer N, Tasic H, Adeyemi O, *et al*. Concordance and determinants of mothers’ and children’s diets in Nigeria: an in-depth study of the 2018 Demographic and Health Survey. *BMJ Open* 2023;13:e070876. doi: 10.1136/bmjopen-2022-070876

This article was previously published with an error.

Figure 2 contains an error. Rather than presenting different findings for three age groups, the results were duplicated three times for a single age group. The corrected figure is displayed below for your reference.

Figure 2 (A) Percentage concordance and discordance between maternal and child consumption of specific food groups in past 24 hours. (B) Percentage concordance between maternal and child consumption of specific food groups in past 24 hours (child age 6–11 months). (C) Percentage concordance between maternal and child consumption of food groups (child age 12–23 months).

**Figure:**