# Supplementary material for: Concordance and determinants of mothers’ and children’s diets in Nigeria: an in-depth study of the 2018 Demographic and Health Survey
Source: BMJ Open. 2023 Jul 11;13(7):e070876. doi: 10.1136/bmjopen-2022-070876 (PMC10347484; doi:10.1136/bmjopen-2022-070876)

Supplementary Documents

Supplementary Figure 1: Modified UNICEF Framework showing distal, intermediate and proximal determinants of inadequate dietary intake for children

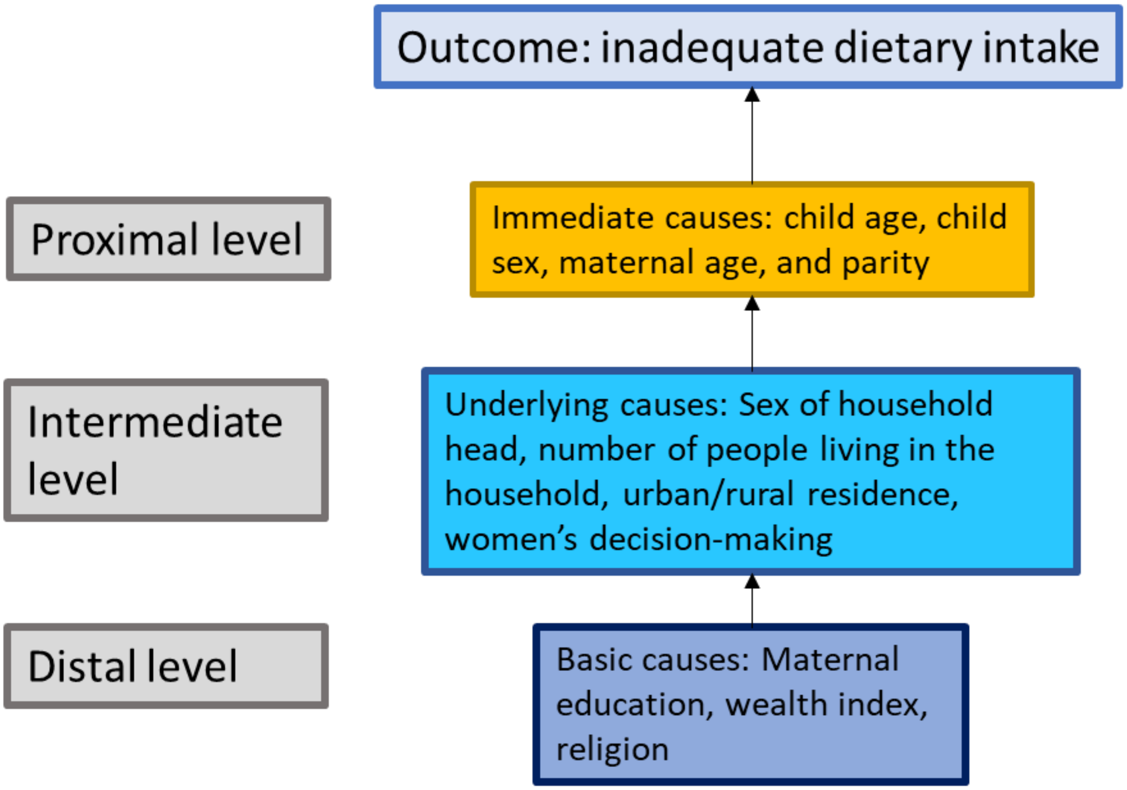

Figure 2A: Percentage concordance between maternal and child consumption of food groups (**adolescent mothers age 15-19 years**)

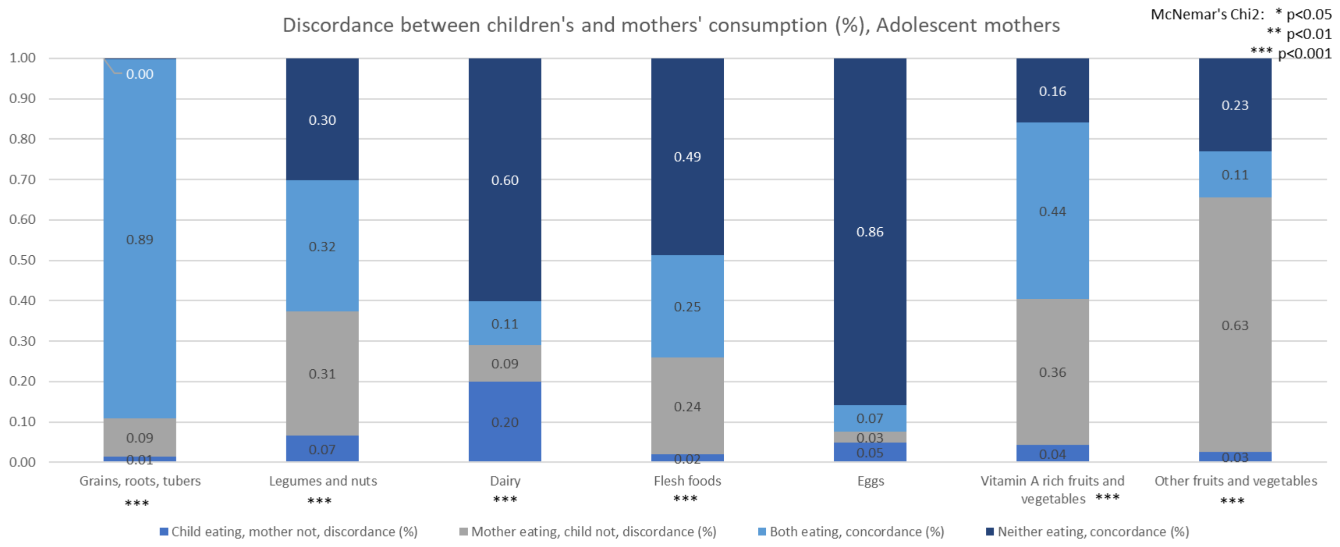

Figure 2B: Percentage concordance between maternal and child consumption of food groups (**older mothers age 35+ years**)

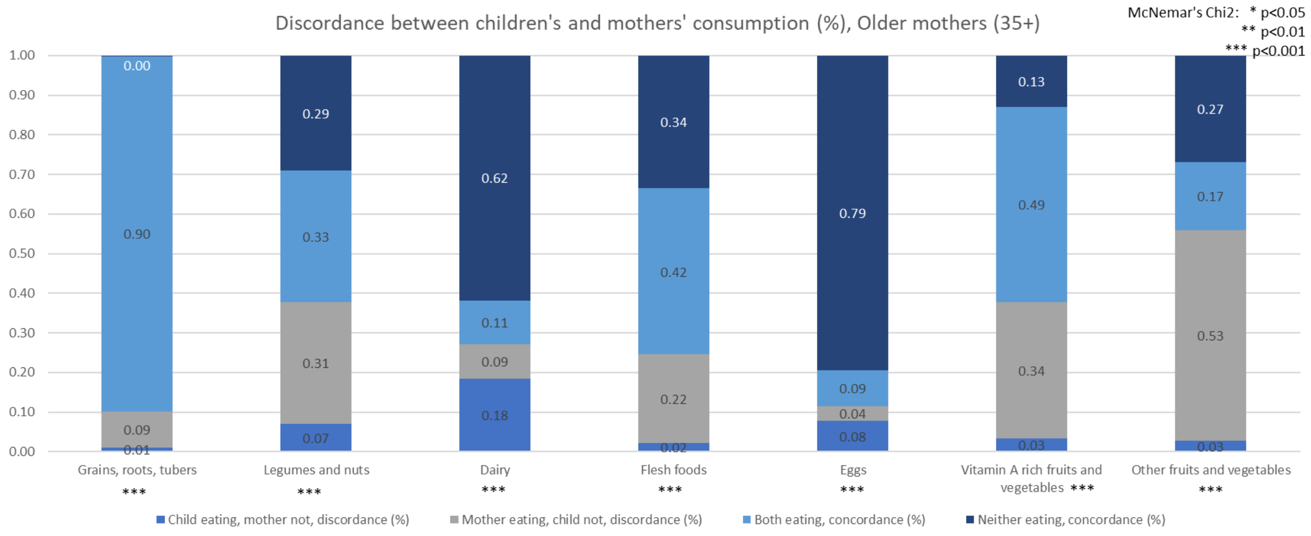

Figure 3A: Percentage concordance between maternal and child consumption of food groups (**urban residence**)

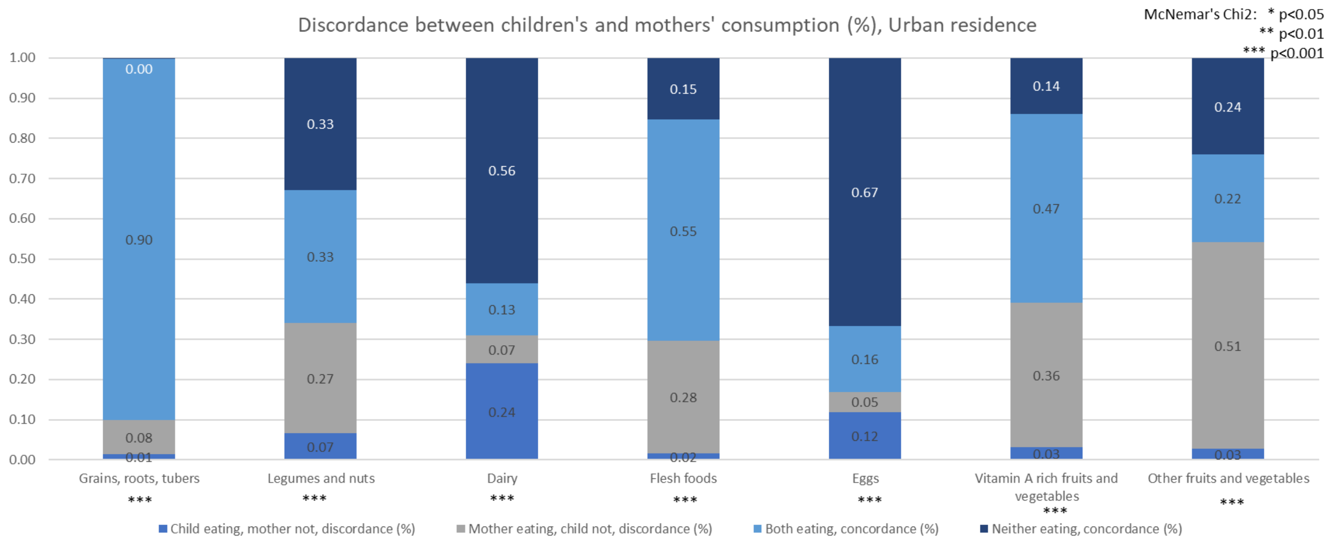

Figure 3B: Percentage concordance between maternal and child consumption of food groups (**rural residence**)

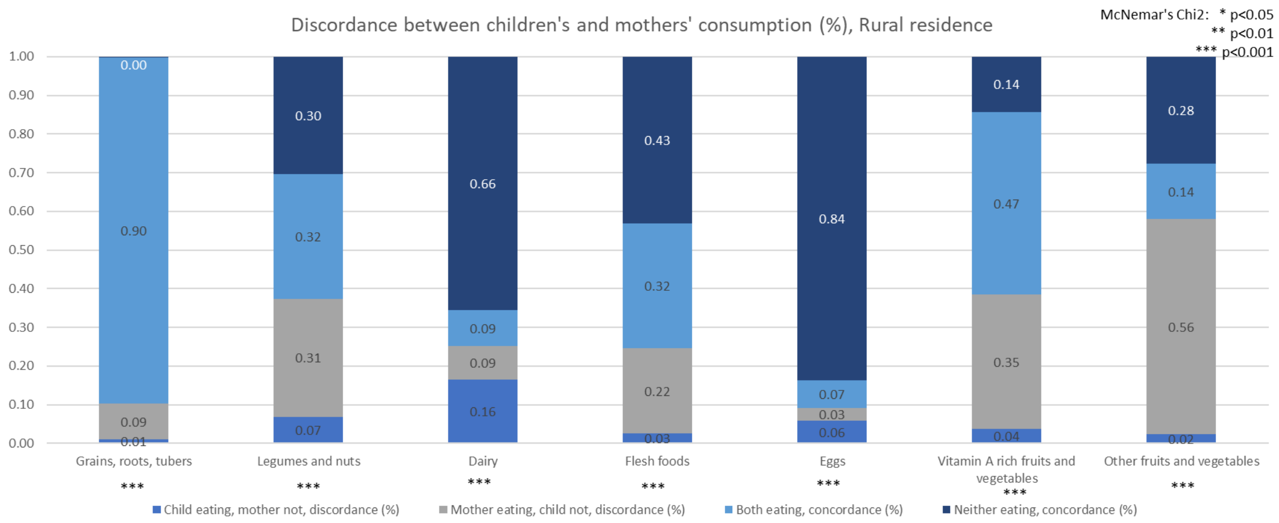

Figure 4A: Percentage concordance between maternal and child consumption of food groups (**no education**)

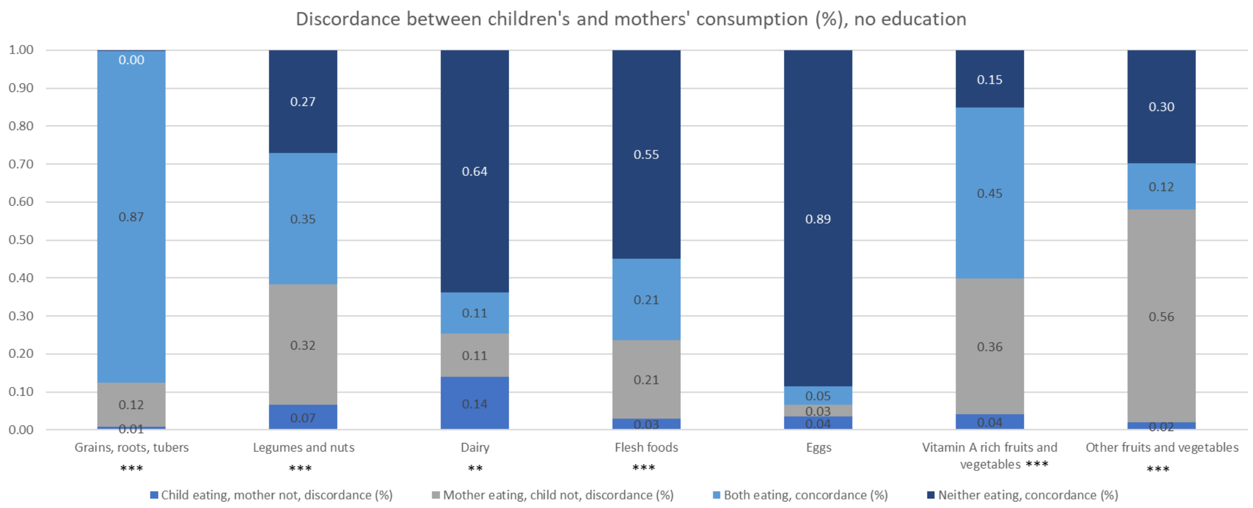

Figure 4B: Percentage concordance between maternal and child consumption of food groups (**any education**)

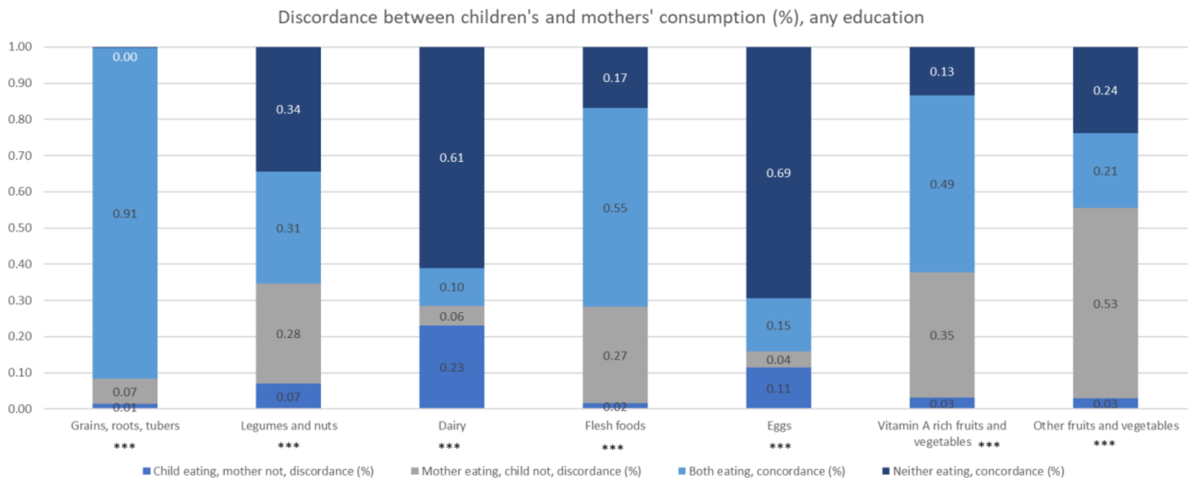

Figure 5A: Percentage concordance between maternal and child consumption of food groups (**bottom two quintiles**)

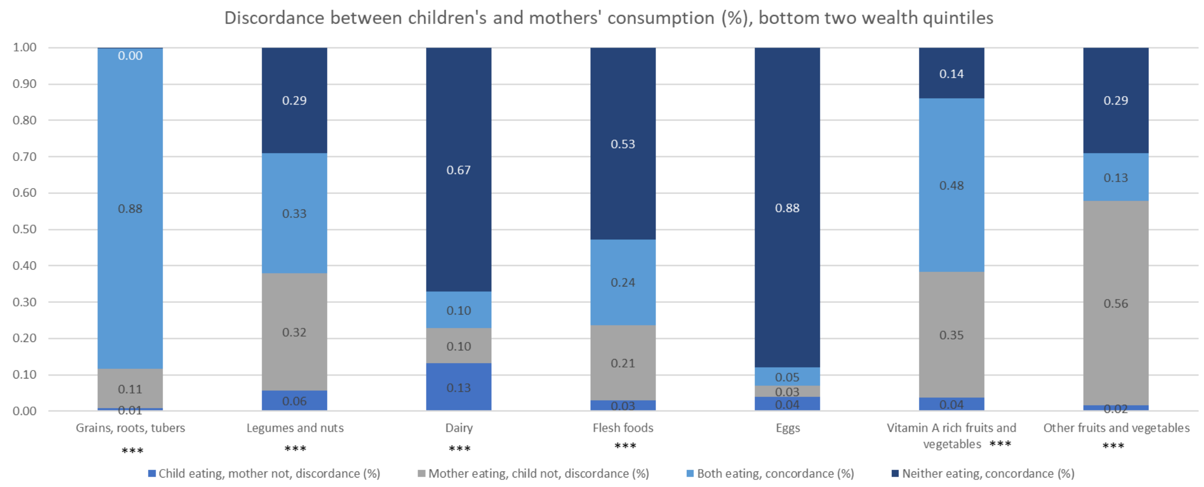

Figure 5B: Percentage concordance between maternal and child consumption of food groups (**upper three quintiles**)

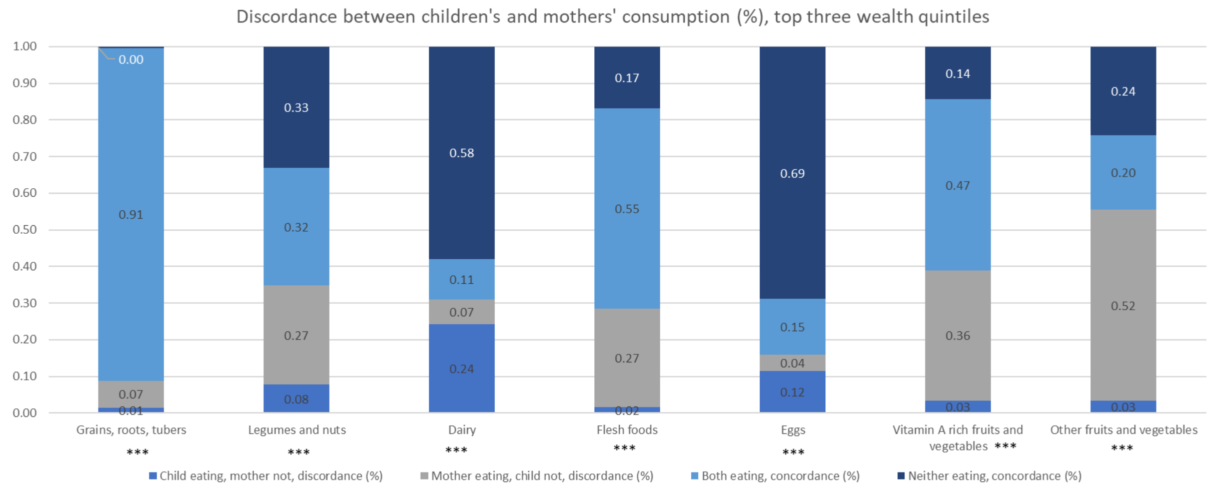

Figure 6: Percentage concordance between maternal and child achievement of minimum dietary diversity

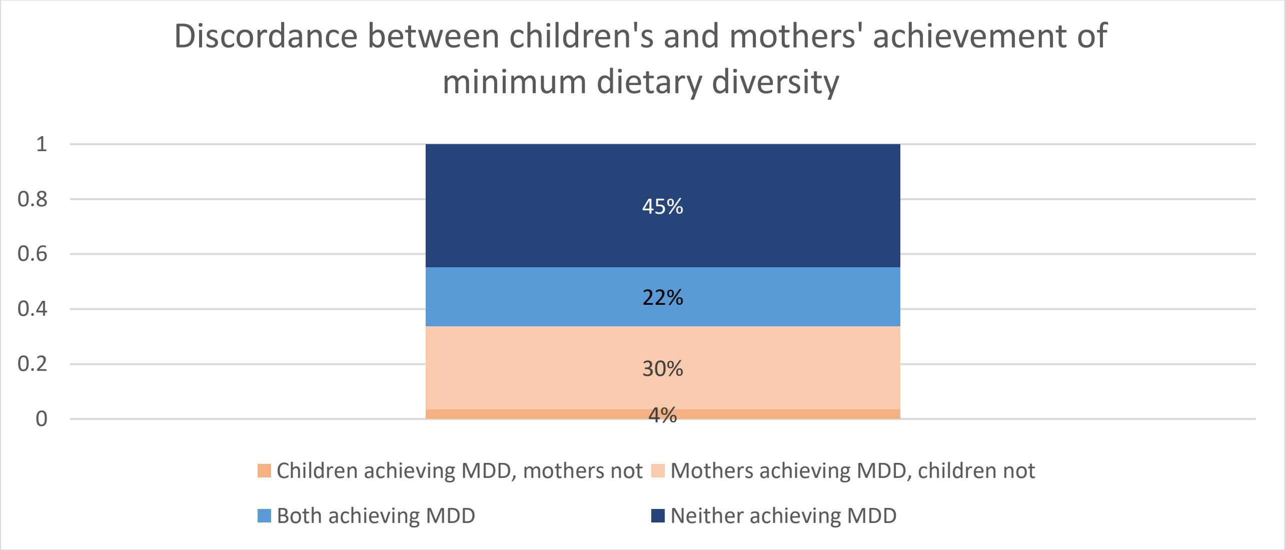

Figure 7A: MDD-W crude and adjusted impact on child breastmilk intake in the past 24 hours

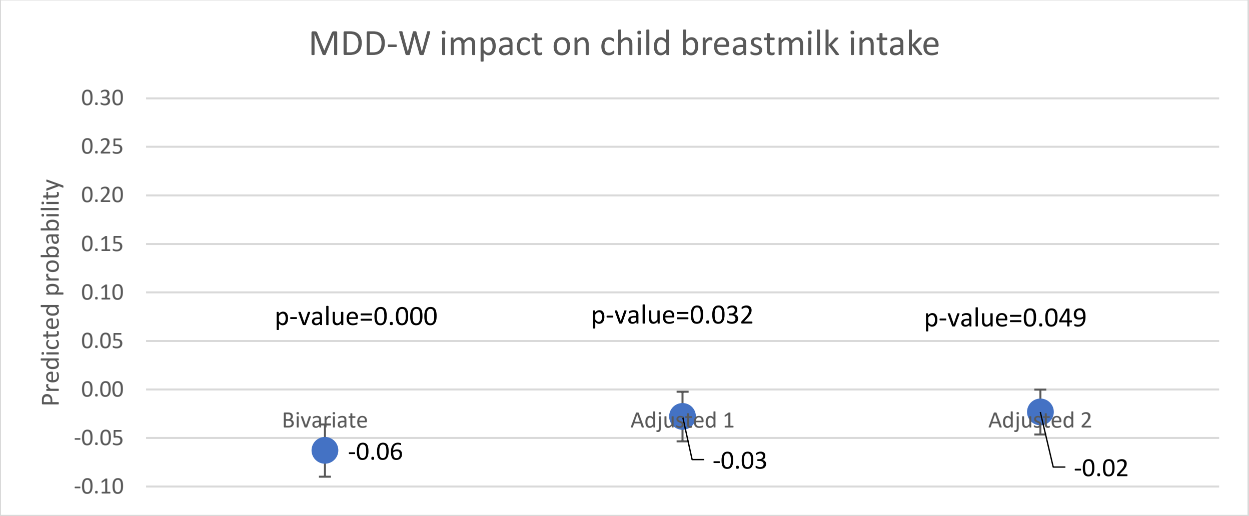

Note: Adjusted 1 is adjusted for distal/intermediate variables: Wealth index, Maternal education, Place of residence (urban/rural); Adjusted 2 is adjusted for Proximal variables: Child age, Child sex, Maternal age, parity

Figure 7B: MDD-W crude and adjusted impact on child grains, roots and tubers intake in the past 24 hours

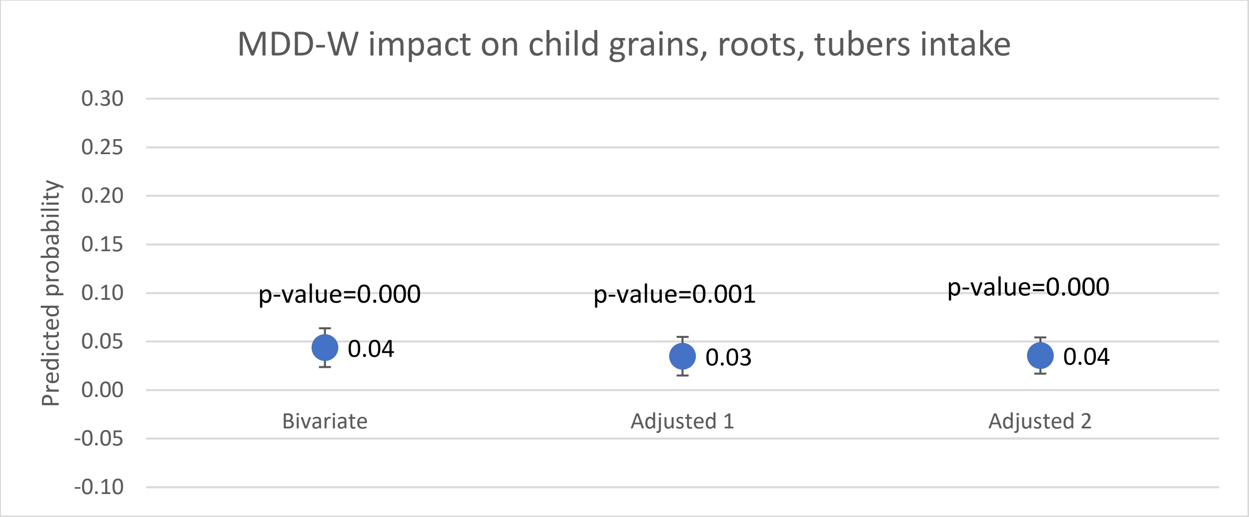

Figure 7C: MDD-W crude and adjusted impact on child legumes and nuts intake in the past 24 hours

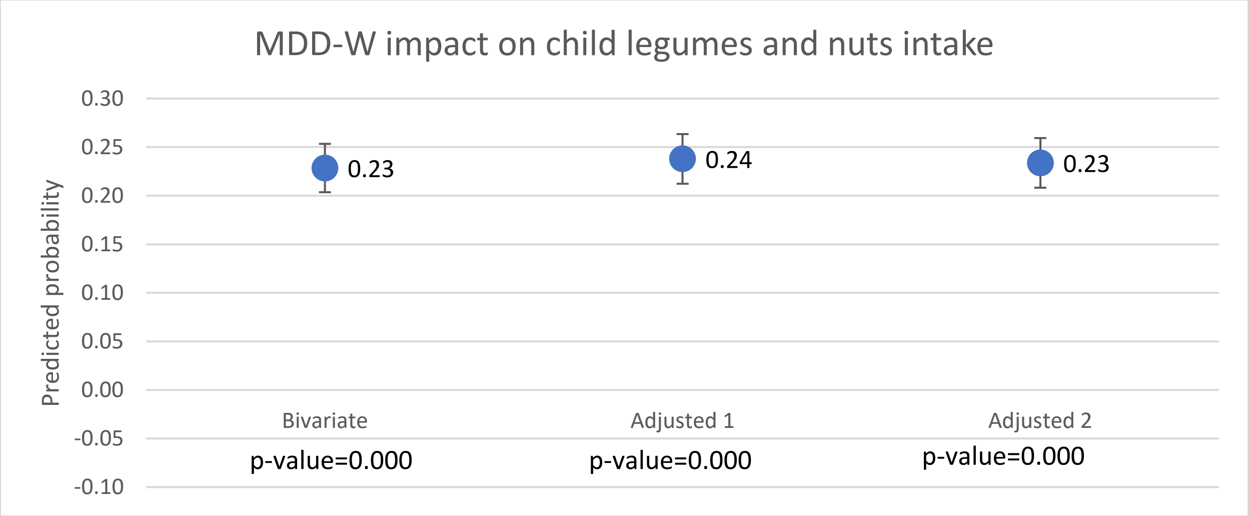

Figure 7D: MDD-W crude and adjusted impact on child dairy products intake in the past 24 hours

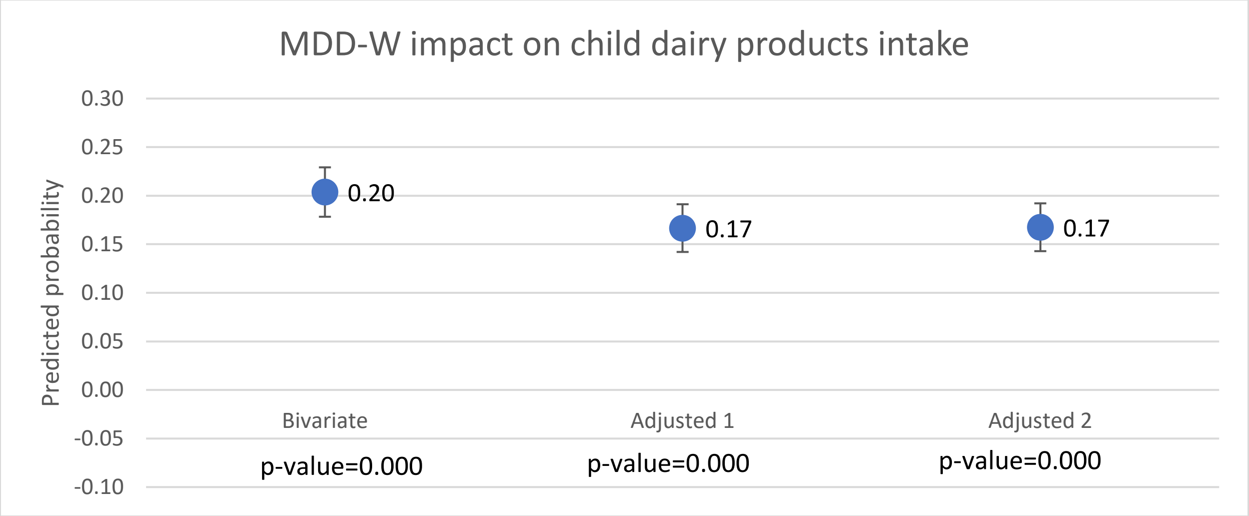

Figure 7E: MDD-W crude and adjusted impact on child flesh foods intake in the past 24 hours

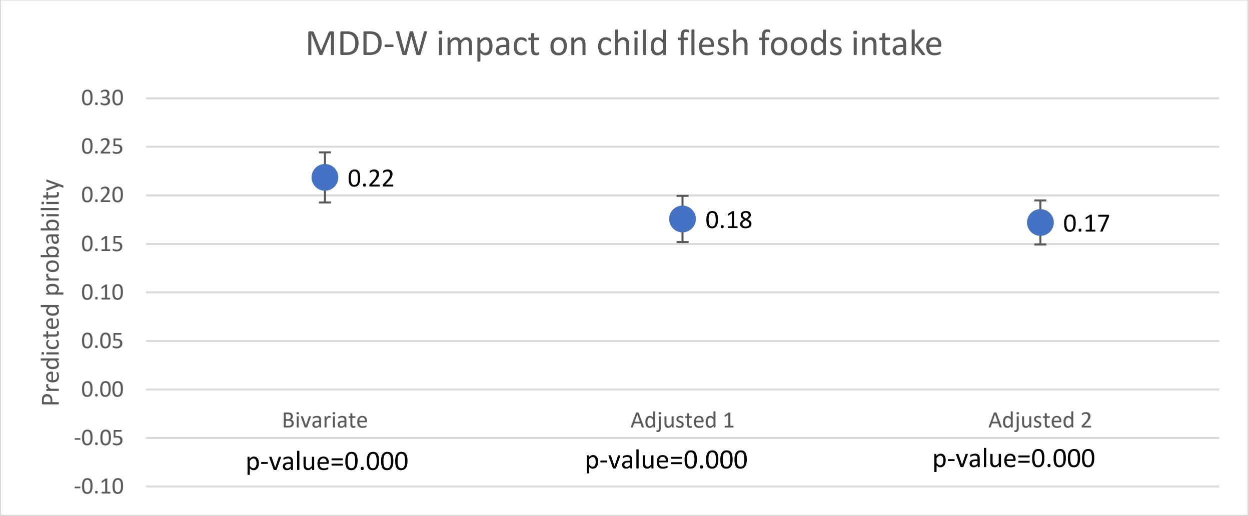

Figure 7F: MDD-W crude and adjusted impact on child egg intake in the past 24 hours

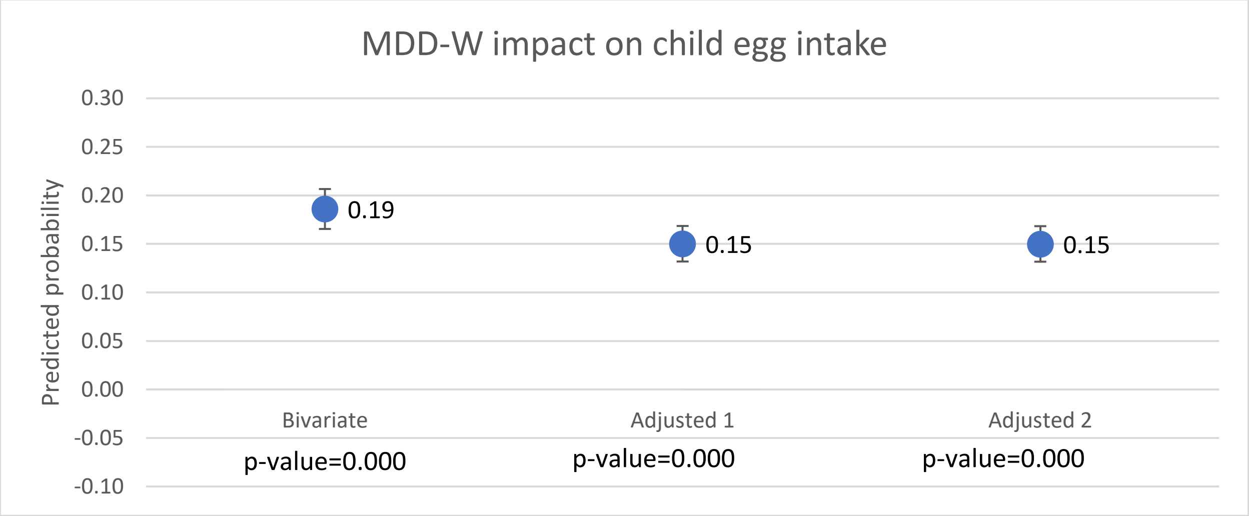

Figure 7G: MDD-W crude and adjusted impact on child vitamin A rich fruits and vegetables intake in the past 24 hours

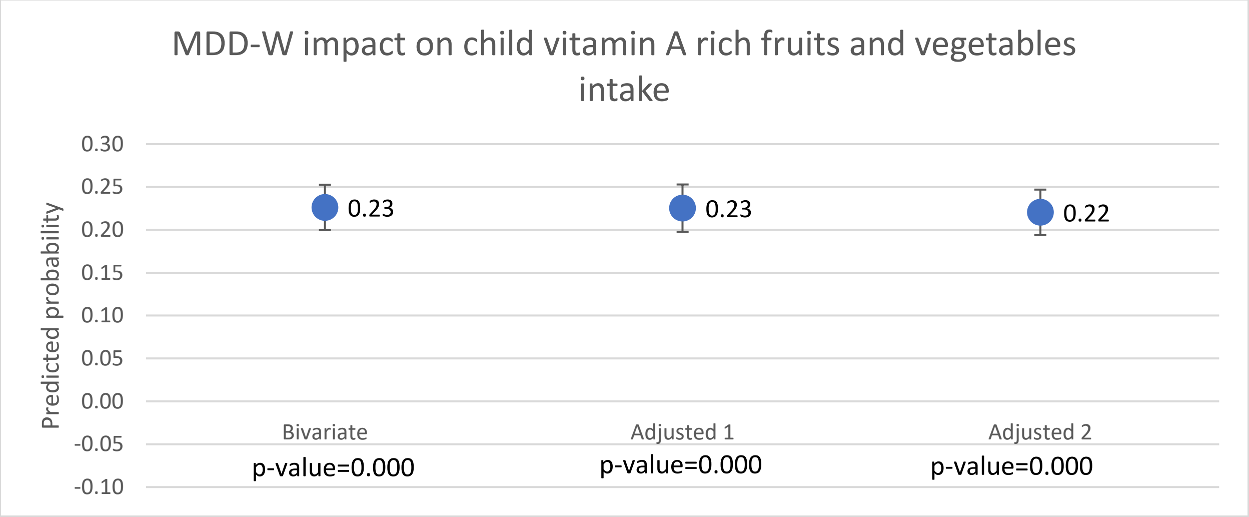

Figure 7H: MDD-W crude and adjusted impact on child other fruits and vegetables intake in the past 24 hours

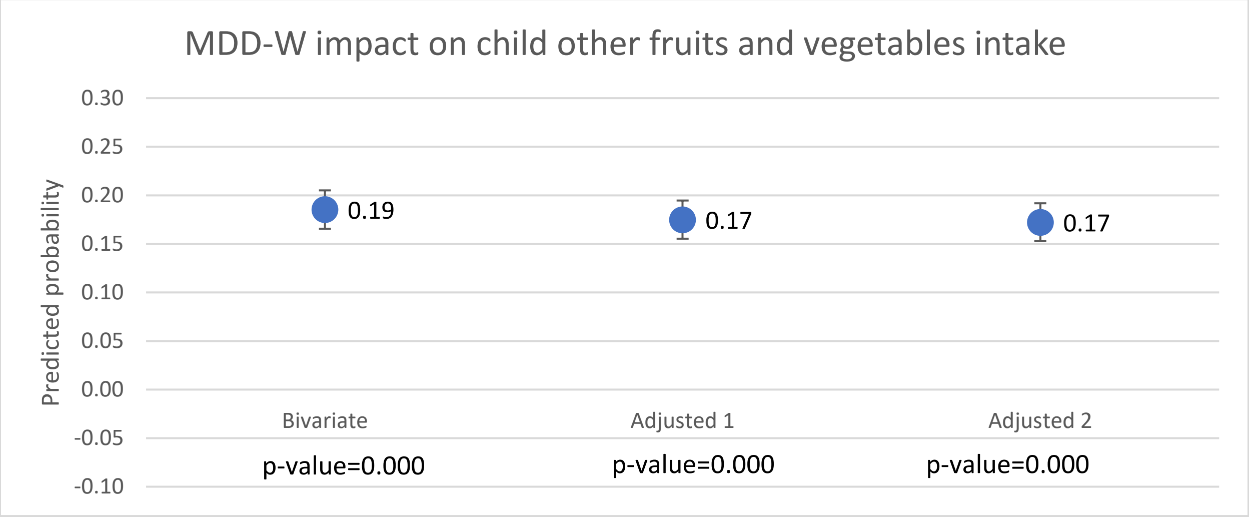

Figure 7I: MDD-W crude and adjusted impact on child minimum diet diversity in the past 24 hours

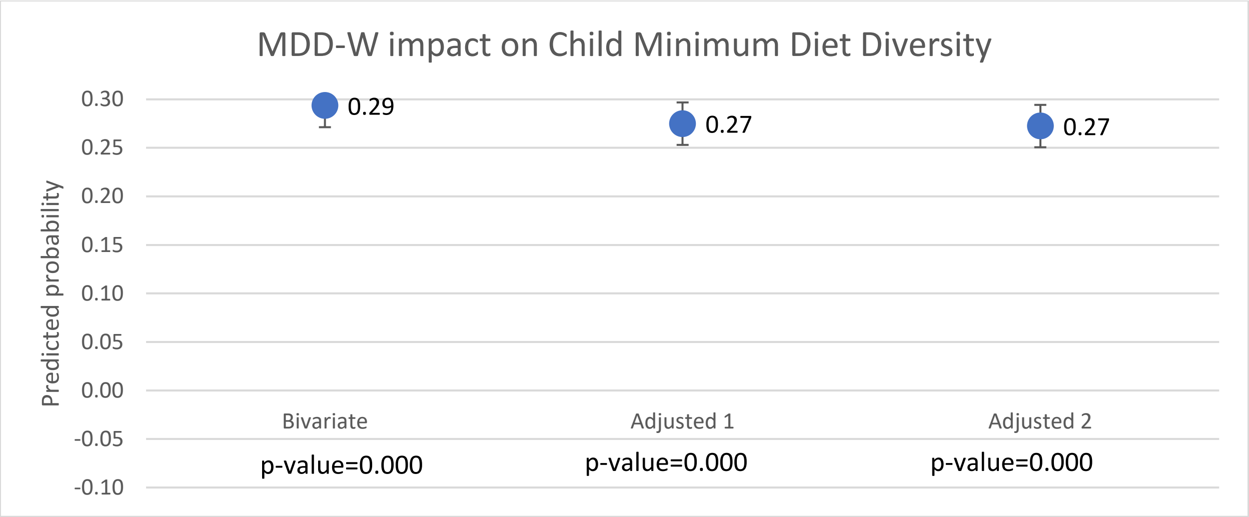

Supplement: online supplemental file 1 [file bmjopen-2022-070876supp001.pdf]
