# Supplementary material for: Concordance and determinants of mothers’ and children’s diets in Nigeria: an in-depth study of the 2018 Demographic and Health Survey
Source: BMJ Open. 2023 Jul 11;13(7):e070876. doi: 10.1136/bmjopen-2022-070876 (PMC10347484; doi:10.1136/bmjopen-2022-070876)
Supplement: Supplementary file 2 [file bmjopen-2022-070876.reviewer_comments.pdf]

PEER REVIEW HISTORY

BMJ Open publishes all reviews undertaken for accepted manuscripts. Reviewers are asked to complete a checklist review form (**http://bmjopen.bmj.com/site/about/resources/checklist.pdf**) and are provided with free text boxes to elaborate on their assessment. These free text comments are reproduced below.

ARTICLE DETAILS

**TITLE (PROVISIONAL)**	Concordance and determinants of mothers' and children's diets in Nigeria: an in-depth study of the 2018 Demographic and Health Survey
**AUTHORS**	Akseer, Nadia; Tasic, Hana; Adeyemi, Olutayo; Heidkamp, Rebecca

VERSION 1 – REVIEW

**REVIEWER**	Azevedo , Francilene<br>Universidade Federal de Viçosa, Departamento de Nutrição e Saúde
**REVIEW RETURNED**	02-Feb-2023

**GENERAL COMMENTS**	In general, the text is well written, the methodology is robust and the results are sufficient to show the relationship between maternal and child consumption.<br><br>1- I believe that the work is of great relevance and only needs minor adjustments to adapt to the English language.<br><br>2 - The last two paragraphs of the introduction contain information that should be included in the methodology and conclusions, respectively.<br><br>3 - I suggest inserting the objective at the end of the last paragraph of the introduction.<br><br>4 - The methodology is well written, however, I suggest including an explanation about the chosen subsample (Our analyses were restricted to 8,975 pairs of mothers aged 15-49 years and their children aged 6-23 months).<br><br>5. In the discussion, some paragraphs are long, I suggest making them more objective.

**REVIEWER**	Mohammed, Shimels Hussien<br>Tehran University of Medical Sciences
**REVIEW RETURNED**	17-Apr-2023

**GENERAL COMMENTS**	The manuscript is done on a relevant topic and used nationally representative large data. I recommend accepting the manuscript after addressing the following minor comments.<br>1) Include 95% confidence intervals with their corresponding p-values at the abstract and other relevant sections.<br>2) Present the level of concordance in percentage figures by MDD-c and MDD_w and common food groups. What percent of mother-

	child dyads meet both MDD-w and MDD-w?<br>3) Define abbreviations at each table

VERSION 1 – AUTHOR RESPONSE

Reviewer	Comment	Response
Reviewer 1	I believe that the work is of great relevance and only needs minor adjustments to adapt to the English language.	Thank you very much!
	The last two paragraphs of the introduction contain information that should be included in the methodology and conclusions, respectively.	OK, these have been moved to the methodology and conclusions sections.
	I suggest inserting the objective at the end of the last paragraph of the introduction.	OK, this was included.
	The methodology is well written, however, I suggest including an explanation about the chosen subsample (Our analyses were restricted to 8,975 pairs of mothers aged 15-49 years and their children aged 6-23 months).	The subsample consists of the mother-child dyads that fit the age criteria. We have re-written the sentence to: "Our analyses were restricted to pairs of mothers aged 15-49 years and their children aged 6-23 months, for a total of 8,975 dyads". Hopefully this is clearer.
	In the discussion, some paragraphs are long, I suggest making them more objective.	We have shortened some of the longer paragraphs to make the information more digestible.
Reviewer 2	Include 95% confidence intervals with their corresponding p-values at the abstract and other relevant sections.	OK, this has been added.
	Present the level of concordance in percentage figures by MDD-c and MDD_w and common food groups. What percent of mother-child dyads meet both MDD-w and MDD-w?	This figure has been included in Appendix Figure 6.
	Define abbreviations at each table	OK, abbreviation definitions have been added.
Editor	Please ensure that your abstract is formatted according to our Instructions for Authors: http://bmjopen.bmj.com/pages/authors/#research	Additional sections have been added: setting, participants, primary and secondary outcome measures
	Please remove your 'key messages' section. Instead, please add a section entitled 'Strengths and limitations of this study', immediately after the abstract. This section should contain up to five short bullet points, no longer than one sentence each, that relate specifically to the methods. The novelty, aims, results or expected impact of the study should not be summarised here.	The key messages section has been removed and the Strengths and limitations section has been added.
